# Cathodic Aromatic C,C Cross-Coupling Reaction via Single Electron Transfer Pathway

**DOI:** 10.3390/molecules22030413

**Published:** 2017-03-07

**Authors:** Yang Qu, Hiroyuki Tateno, Yoshimasa Matsumura, Tsuneo Kashiwagi, Mahito Atobe

**Affiliations:** Graduate School of Environment and Information Sciences, Yokohama National University, 79-7, Tokiwadai, Hodogaya-ku, Yokohama 240851, Japan; qu-yang-rv@ynu.jp (Y.Q.); tateno-hiroyuki@aist.go.jp (H.T.); y.matsumura@yz.yamagata-u.ac.jp (Y.M.); tsuneo.kashiwagi@konicaminolta.com (T.K.)

**Keywords:** electrochemical synthesis, single electron transfer, C,C cross-coupling

## Abstract

We have successfully developed a novel cathodic cross-coupling reaction of aryl halides with arenes. Utilization of the cathodic single electron transfer (SET) mechanism for activation of aryl halides enables the cross-coupling reaction to proceed without the need for any transition metal catalysts or single electron donors in a mild condition. The SET from a cathode to an aryl halide initiates a radical chain by giving an anion radical of the aryl halide. The following propagation cycle also consists entirely of anion radical intermediates.

## 1. Introduction

Biaryl derivatives are considered to be useful organic compounds in industry and in the medical field [1]. Owing to their wide usefulness, convenience, and efficient synthetic methods, biphenyl compounds have attracted considerable interest in recent years [2]. The aromatic C,C cross-coupling reaction is one of the most efficient methods for the synthesis of biaryls [3,4,5,6,7]. However, these reactions generally require complex, toxic, and expensive transition metal catalysts. Therefore, the development of an aromatic C,C cross-coupling reaction without the need for any transition metal catalysts is an attractive research target from both fundamental and practical points of view [8,9,10].

The electrochemical approach for the aromatic C,C cross-coupling is economically and environmentally attractive because only electrons serve as reagents and the reaction proceeds under a mild condition [11,12]. Although a number of aromatic C,C cross-coupling reactions by electrochemical method have been developed so far, most of these studies were based on the anodic oxidation process [13,14,15,16,17,18,19]. Anodic oxidation in the mixture of two aromatic compounds can provide cross-coupling products, but their selectivities are usually low [16,17,18,19]. In these cases, the homo-coupling reactions also occur by the non-selective oxidation of the starting materials. In addition, the overoxidation of the cross-coupling products is usually unavoidable during the reaction, because the oxidation potentials of the cross-coupling products (such as biaryl compounds) are generally lower than those of the corresponding substrates. On the other hand, the cathodic cross-coupling reaction for the biphenyl synthesis has been rarely limited so far [20]. Therefore, the development of an efficient cathodic cross-coupling reaction is attractive, and further investigation is still a challenging target.

Shirakawa and his co-workers have recently developed transition-metal-free coupling reactions of aryl halides with arenes [21,22]. These reactions are considered to proceed via pseudo S_RN_1 mechanism and are useful for the synthesis of a variety of biaryl derivatives. However, the stoichiometric amount of a single electron donor (reductant) and high temperature conditions are required to activate aryl halides in the initial step of this reaction, and hence it remains desirable to conduct the aromatic C,C cross-coupling reactions in the absence of an added single electron donor under mild conditions. To overcome these drawbacks, we envisioned that the cathodic reduction would be applicable for the activation of aryl halides in the initial step. By this operation, an aromatic C,C cross-coupling reaction that requires neither transition metal catalyst nor single electron donor would be developed. In addition, the reaction would be conducted at ambient temperature.

**Scheme 1:**
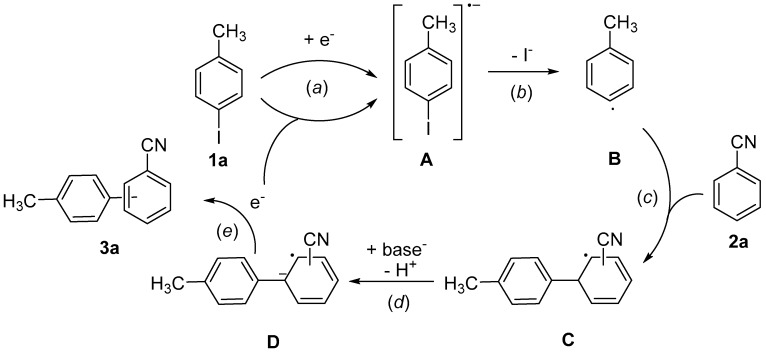
Assumed mechanism for the cathodic cross-coupling reaction of 4-iodotoluene (**1a**) with benzonitrile (**2a**) via single electron transfer (SET) pathway.

Herein, we wish to report the realization of cathodic cross-coupling reactions of aryl halides with arenes via a single electron transfer (SET) pathway. In this demonstration, we chose 4-iodotoluene (**1a**) and benzonitrile (**2a**) as a model aryl halide and arene, respectively. By using these model substrates, we assumed the reaction mechanism for the cathodic aromatic C,C cross-coupling as shown in Scheme 1.

Radical anion **A**, produced by SET from cathode in the initial step (step *a*), would be converted into the aryl radical **B** upon elimination of I^−^ (step *b*). After addition of the aryl radical **B** to benzonitrile **2a** to give the radical intermediate **C** (step *c*), the radical anion **D** is generated by deprotonation with a base (step *d*). SET from the resulting **D** to 4-iodotoluene **1a** gives the coupling product **3a** and regenerates **A** (step *e*).

## 2. Results and Discussion

**Figure 1:**
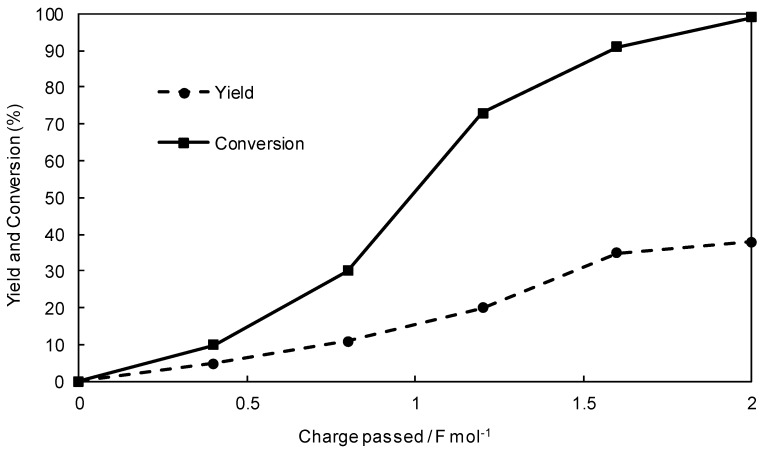
Effect of the electricity on the yield of **3a** and the conversion of **1a**.

**Figure 2:**
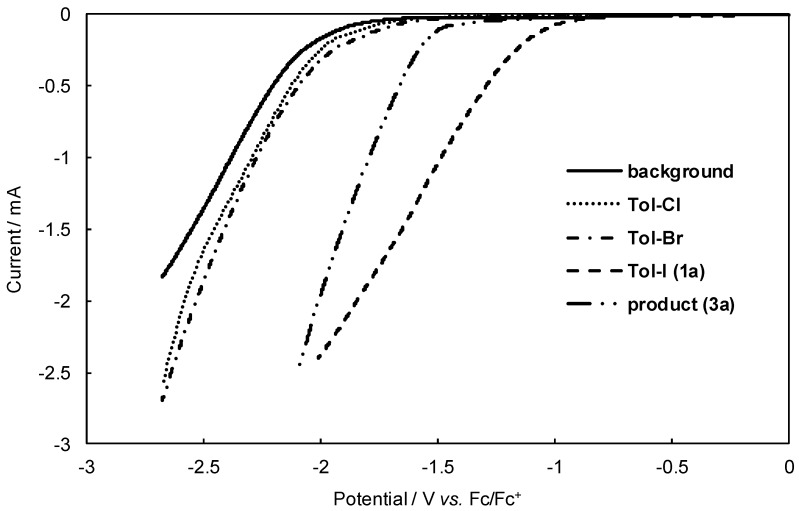
Linear sweep voltammograms of 4-chlorotoluene (**Tol-Cl**), 4-bromotoluene (**Tol-Br**), 4-iodotoluene (**1a**), and the cross-coupling product (**3a**) in *N,N*-dimethylformamide (DMF) electrolytic solution at a scan rate of 0.1 V·s^−1^.

At first, we investigated the effect of the electricity on the yield of **3a** and conversion of **1a** in the cathodic cross-coupling reaction. As shown in Figure 1, the conversion of **1a** was increased with an increase in the electricity, and the starting substrate **1a** was found to be almost consumed at 2 F·mol^−1^. On the other hand, the yield of **3a** was also increased and saturated at 2 F·mol^−1^. However, the yield was relatively low compared to the conversion. This can be ascribed to the over-reduction of aryl radical **B**. In fact, a large amount of over-reduction products such as a toluene was detected by the HPLC analysis. In addition, it was also found that the desired cross-coupling product **3a** was obtained as a regioisomeric mixture (o:m:p = 53:0:47). In this case, however, meta-isomer was not detected at all. The lower reduction potential of **3a** is also problematic for the desired reaction. As shown in Figure 2, the reduction potential of product **3a** is more positive than that of **1a**. Therefore, the over-reduction of **3a** might occur during the reaction. This may be another reason that the conversion yield of the product **3a** was low.

The generation of radical anion **A** (step *a* in Scheme 1) would be influenced by the reduction potential of the starting aryl halide. Hence, in the following, we also used other aryl halides such as 4-chlorotoluene (**Tol-Cl**) and 4-bromotoluene (**Tol-Br**) (Table 1). As shown in Table 1, the coupling reaction with **Tol-Br** and **Tol-Cl** resulted in lower yields compared to the case with **1a**. This can be ascribed to the reduction potential of the starting aryl halides; that is, the use of **Tol-Br** and **Tol-Cl** would generate the radical anion **A** with more difficulty due to their higher reduction potentials. To confirm the reduction potential of the starting aryl halides, we then measured their linear sweep voltammograms (Figure 2). Indeed, **1a** exhibited much lower reduction potential than other aryl halides (**Tol-Br** and **Tol-Cl**).

**Figure 3:**
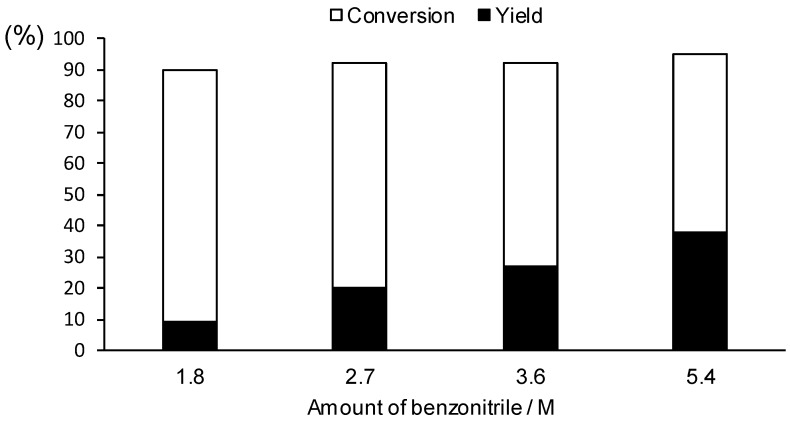
Effect of the benzonitrile (**2a**) concentration on the yield of **3a** and conversion of **1a**.

Subsequently, the effect of the concentration of benzonitrile (**2a**) in the electrolytic solution was investigated. As shown in Figure 3, the yield of the cross-coupling product **3a** increased with an increase in the concentration of **2a**, although the conversion was almost constant (more than 90%), regardless of the **2a** concentration. This can be ascribed to the fact that the aryl radical **B** was effectively trapped by the high concentration of **2a** (step *c* in Scheme 1) before it was further reduced.

Furthermore, we also carried out the cathodic cross-coupling reaction without a base in order to check the necessity of a base in step *d* of Scheme 1. As shown in Entry 1 of Table 2, no cross-coupling reaction took place without a base, while the desired cross-coupling reaction proceeded with all kinds of bases (Entries 2–4). Therefore, the deprotonation from the radical anion **D** (step *d*) might be an essential step for the cathodic cross-coupling reaction. On the other hand, the effect of the type of bases used in this work was found to be less remarkable.

**Figure 4:**
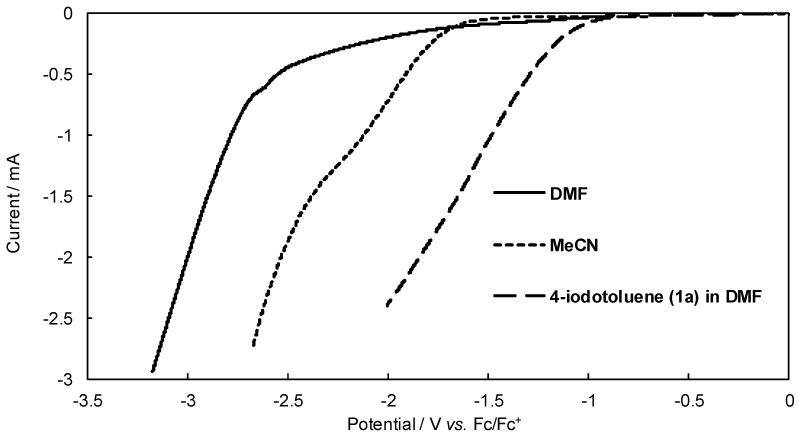
Linear sweep voltammograms of DMF electrolytic solutions with and without **1a**, and acetonitrile electrolytic solution without **1a** at a scan rate of 0.1 V·s^−1^.

In the next, we investigated the effect of the current density on the yield of **3a** and conversion of **1a** (Entries 2, 5–7 of Table 2). Although the conversion was almost constant (more than 90%) at all current densities (15 to 35 mA·cm^−2^), the yield decreased at current densities higher than 30 mA·cm^−2^. This may be ascribed to the over-reduction of aryl radical **B** and/or the product **3a**. Therefore, 20 mA·cm^−2^ seems to be the optimum current density for the cross-coupling reaction. The effect of the reaction temperature was also investigated (Entries 2, 8, and 9). We expected that step *c* proceeds faster than the over-reduction of aryl radical **B** at higher reaction temperatures. However, the yield of **3a** was influenced little by the reaction temperature. Furthermore, the reaction was also carried out in an acetonitrile solution (Entry 10). Not only the yield of **3a**, but also the conversion of **1a** decreased in this case. This can be ascribed to the potential window of solvents used in this work. As shown in Figure 4, discharge of acetonitrile takes place more positive than that of DMF. Therefore, this may interfere with the cathodic reduction of the starting substrate **1a**. Therefore, it can be stated that DMF is a better electrolytic solvent in this reaction.

In the next, to demonstrate the general versatility, we carried out the cathodic cross-coupling reaction with various substrates (Table 3). Similar to the case with the arene **2a** (Entry 1), the cross-coupling reaction of 4-iodotoluene (**1a**) with an arene having an electron-donating group (**2b**) also provided the corresponding coupling product **3b** in a reasonable yield (Entry 2). As shown in Entries 3 and 6, the yields of the coupling products increased to some extent when phenyl iodides having an electron-withdrawing group (**1b** and **1c**) were used as starting aryl halides. This might be ascribed to the fact that phenyl iodides having an electron-withdrawing group were more reactive than **1a**. On the other hand, the cross-coupling reaction of 4-iodotoluene (**1a**) with 1,4-dimethoxybenzene (**2c**) also proceeded to give the product **3d** in a moderate yield (Entry 4), while the reaction with 1,3,5-trimethoxybenzene resulted in a lower yield of **3e** due to its steric hindrance (Entry 5).

**Figure 5:**
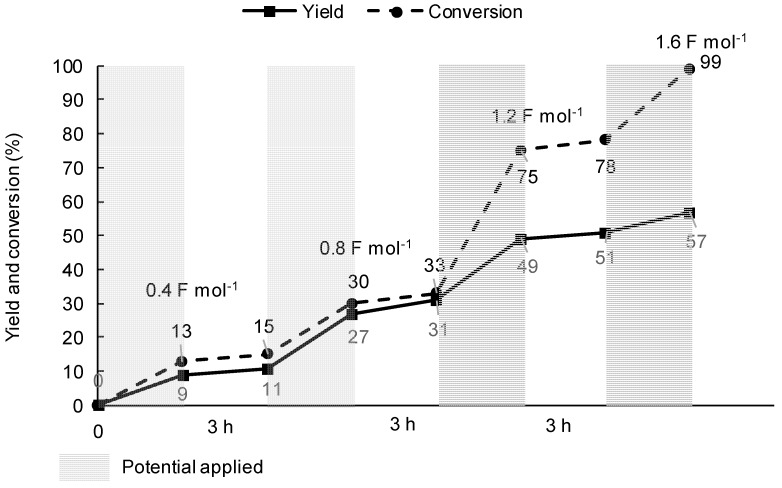
Yield of **3a** and conversion of **1a** on the interval-electrolysis.

According to our assumed mechanism shown in Scheme 1, step *e* is essential for giving the coupling product **3a**. In addition, this step would also involve spontaneous initiation for the coupling reaction because it regenerates the radical anion **A**. Therefore, it can be expected that the coupling reaction proceeds even if catalytic amounts of electricity are provided on the starting aryl halide **1a**. To confirm this, we then monitored the yield and conversion variation with time after the electrolysis with 1 F·mol^−1^. Certainly, we carried out this electrolysis under argon atmosphere in order to avoid the impact of oxygen. As shown in Table 4, both yield and conversion increased gradually with increasing time after stopping the electrolysis. Hence, as hypothesized, it was confirmed that step *e* was essential for the catalytic SET process in the cross-coupling reaction. In addition, it should be noted that an increased amount of the conversion was almost the same as that of the yield of **3a** after stopping the electrolysis, and the over-reduction products such as a toluene were mostly not produced during this period. However, both yield and conversion were almost saturated at 3 h. Hence, a further application of the electricity is necessary to restart the coupling reaction in this situation. From these facts, in order to conduct an efficient catalytic SET process for the cross-coupling, we conceived of the stair-step application of electricity—namely, interval-electrolysis. This would be useful to reduce the electricity applied and increase the selectivity for the coupling product. Indeed, the amount of the coupling product at each stage increased with time—even when no electricity was applied, as shown in Figure 5. In addition, it is also noted that 4-iodotoluene (**1a**) was almost consumed with less electricity (total electricity applied, 1.6 F·mol^−1^), and the yield of the coupling product **3a** increased by up to 57%. This may be ascribed to the fact that an efficient catalytic SET in the cross-coupling reaction occurred when no electricity was applied, and hence, the toluene formation was suppressed in this demonstration.

## 3. Materials, Instruments, and Methods

### 3.1. Materials and Instruments

Anhydrous *N,N*-dimethylformamide (DMF) and acetonitrile were obtained from Kanto Chemical Cp., Inc., Tokyo, Japan, and used as received. 4-iodotoluene (**1a**), 4-bromotoluene (**Tol-Br**), 4-chlorotoluene (**Tol-Cl**), benzonitrile (**2a**), sodium *tert*-butoxide, tetrabutylammonium tetrafluoroborate (TBABF_4_), 1-cyano-4-iodobenzene (**1b**), 4-chloro-iodobenzene (**1c**), anisole (**2b**), 1,4-dimethoxybenzene (**2c**), 1,3,5-trimethoxybenzene (**2d**), ferrocene, and nitromethane were obtained from Tokyo Chemical Industry and used as received. Distilled water was prepared by automatic water distillation apparatus (SA-2100E1, Tokyo Rikakikai Co., Ltd., Tokyo, Japan). Preparative scale electrolyses were carried out with a HOKUTO DENKO HABF-501A Potentiostat/Galvanostat. Gas chromatography mass spectrometry (GCMS) analyses were performed with a Shimadzu gas chromatograph mass spectrometer (GCMS-QP2010) by using a capillary column (0.40 μm, 40.0 m, 0.189 mmID; DB-1, Agilent Technologies, Inc., Tokyo, Japan). Reverse phase high performance liquid chromatography (RP-HPLC) analyses were performed by using a Shimadzu LC-20AD liquid chromatograph equipped with a UV detector (SPD-10AVi, Shimadzu, Kyoto, Japan) and a column (RP-18GP Aqua 250–4.6 (5 μm), Kanto Chemical, Tokyo, Japan). Linear sweep voltammetry measurements were performed by using a computer-controlled electrochemical analyzer (ALS/CH Instruments 630C, Tokyo, Japan). Nuclear magnetic resonance (^1^H-NMR) spectra were measured on BRUKER DRX 300 spectrometer operating at 300 MHz (^1^H-NMR) in CDCl_3_. All ^1^H-NMR chemical shifts were reported in ppm relative to internal references of tetramethylsilane (TMS) at 0.00.

### 3.2. Linear Sweep Voltammetry Measurements

Linear Sweep Voltammetry was recorded using an undivided cell equipped with a working electrode (Pt disk electrode, φ 3 mm), a counter electrode (Pt wire), and a reference electrode (Ag wire). The ferrocene/ferrocenium couple (Fc/Fc^+^) was also measured in the same electrochemical system, and the electrode potential was reported as values referred to the apparent standard potential of the system. Before measurement of linear sweep voltammograms, electrolytes were bubbled by argon gas for 30 min.

### 3.3. General Procedure for Preparative Scale Electrolysis Using H-Type Divided Cell

Aryl halides **1a**, **1b**, and **1c** were used as substrates, and arenes **2a**, **2b**, **2c**, and **2d** were used as coupling partners for the cathodic cross-coupling reaction. The catholyte was a DMF solution (10 mL) containing a substrate (90 mM, 0.90 mmol), a coupling partner (5.4 M, 54 mmol), NaO*t*-Bu (0.18 M, 1.8 mmol), and tetrabutylammonium tetrafluoroborate (0.10 M, 1.0 mmol). The anolyte was a DMF solution (10 mL) containing tetrabutylammonium tetrafluoroborate (0.10 M, 1.0 mmol). Pt electrodes (1.0 × 1.0 cm^2^) were used as working and counter electrodes. Unless stated otherwise, preparative scale electrolyses were performed by constant current method (20 mA·cm^−2^, 2 F·mol^−1^) at 25 °C. After the reaction, the conversion of **1a** and the yield of product (**3a**) were determined by RP-HPLC. On the other hand, the yields of **3b**, **3c**, **3d**, and **3e** were determined by ^1^H-NMR spectra. In addition, product **3a** was isolated by the following procedure. After electrolysis, the electrolytic solution was repeatedly extracted with chloroform several times, and then evaporated to give a yellow liquid. The liquid was subjected to column chromatography (7:3 acetonitrile/water as the eluent). The final product **3a** was purified by the extraction with diethyl ether, and a white solid was afforded after evaporation (0.0363 g, isolated yield was 21%).

### 3.4. General Procedure for Interval-Electrolysis

The catholyte was a DMF solution (80 mL) containing 4-iodotoluene (90 mM, 7.2 mmol), benzonitrile (5.4 M, 0.43 mol), NaO*t*-Bu (0.18 M, 14 mmol), and tetrabutylammonium tetrafluoroborate (0.10 M, 8.0 mmol). The anolyte was a DMF solution (80 mL) containing tetrabutylammonium tetrafluoroborate (0.10 M, 8.0 mmol). Pt electrodes (2.0 × 2.0 cm^2^) were used as working and counter electrodes. Before the electrolysis, the catholyte and anolyte underwent argon gas bubbling for 30 min. Interval-electrolysis involved alternating electrolysis period and rest period (3 h) as shown in Figure 5. During each electrolysis period, electrolysis was performed at constant current method (20 mA·cm^−2^, 0.4 F·mol^−1^) at 25 °C. The reaction mixture was sampled at appropriate time intervals and subjected to RP-HPLC to determine the yield of cross-coupling products and the conversion of substrate.

## 4. Conclusions

We have developed a novel cathodic cross-coupling reaction of aryl halides with arenes. Utilization of the cathodic SET mechanism for the activation of aryl halides enables the cross-coupling reaction to proceed without the need for any transition metal catalysts and single electron donors in a mild condition. The SET from a cathode to an aryl halide initiates a radical chain by giving an anion radical of the aryl halide. The following propagation cycle also consists entirely of anion radical intermediates. We hope that our results promote the research concerning cathodic aromatic C,C cross-coupling reaction.

## Figures and Tables

**Table 1:**
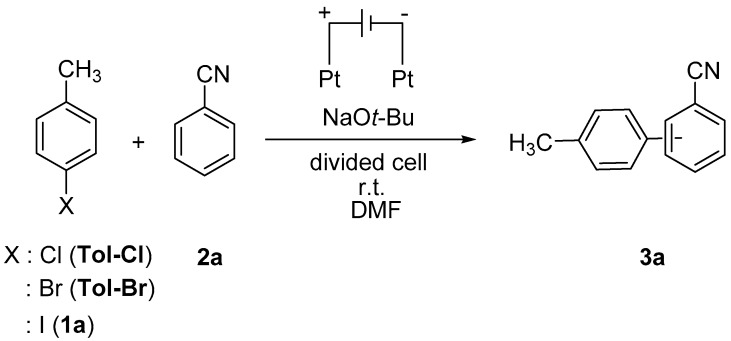
Cathodic cross-coupling reaction using various types of aryl halides.

Entry	Substrate	Yield ^a^ (%)
1	**Tol-Cl**	15
2	**Tol-Br**	26
3	**1a**	38

^a^ Determined by reverse phase (RP)-HPLC. Substrate concentration: 90 mM.

**Table 2:** Cathodic cross-coupling reaction of 4-iodotoluene (**1a**) with benzonitrile (**2a**) in various reaction conditions.

Entry	Base	Current Density/mA·cm^−2^	Reaction Temperature/°C	Yield of 3a ^a^ (%)	Conversion of 1a (%)
1	none	20	25	n.d.	88
2	NaO*t*-Bu	20	25	38	>99
3	pyridine	20	25	35	93
4	trimethylamine	20	25	33	97
5	NaO*t*-Bu	15	25	38	>99
6	NaO*t*-Bu	25	25	35	>99
7	NaO*t*-Bu	30	25	28	>99
8	NaO*t*-Bu	20	40	40	92
9	NaO*t*-Bu	20	80	41	>99
10 ^b^	NaO*t*-Bu	20	25	19	85

Experimental condition: Cathode and anode, Pt plates (1.0 × 1.0 cm^2^); Electricity, 2 F·mol^−1^; catholyte, 90 mM 4-iodotoluene (**1a**) and 5.4 M benzonitrile (**2a**), and 0.18 M base in 0.1 M tetrabutylammonium tetrafluoroborate (TBABF_4_)/DMF; anolyte, 0.1 M TBABF_4_/DMF. ^a^ Determined by RP-HPLC. Substrate concentration: 90 mM. ^b^ The reaction was carried out in an acetonitrile solution (0.1 M TBABF_4_/acetonitrile) instead of DMF solution.

**Table 3:** Cathodic cross-coupling reaction of aryl halides with arenes.

Entry	Aryl Halide	Arene	Cross-Coupling Product	Yield (%)
1	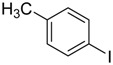 **1a**	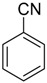 **2a**	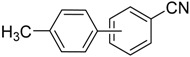 **3a**	38 ^a^ (o:m:p = 53:0:47)
2	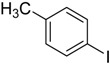 **1b**	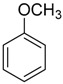 **2b**	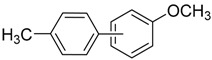 **3b**	47 ^b^ (o:m:p = 42:0:58)
3	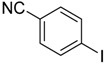 **1b**	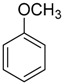 **2b**	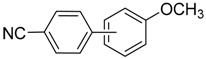 **3c**	52 ^b^ (o:m:p = 0:0:100)
4	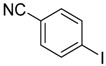 **1b**	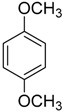 **2c**	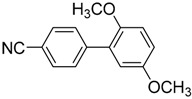 **3d**	42 ^b^
5	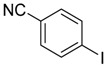 **1b**	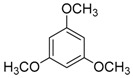 **2d**	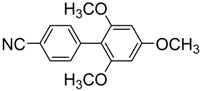 **3e**	11 ^b^
6	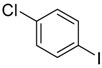 **1c**	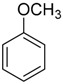 **2b**	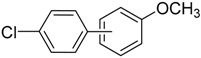 **3f**	49 ^b^ (o:m:p = 0:24:76)

Experimental condition: cathode and anode, Pt plates (1.0 × 1.0 cm^2^); current density, 20 mA·cm^−2^; Electricity, 2 F·mol^−1^; catholyte, 90 mM aryl halide and 5.4 M arene, and 0.18 M NaO*t*-Bu in 0.1 M TBABF_4_/DMF; anolyte, 0.1 M TBABF_4_/DMF; reaction temperature, 25 °C. ^a^ Determined by RP-HPLC. The isolated yield of **3a** in entry 1 is 21% (o:m:p = 62:0:38). ^b^ Determined by ^1^H-NMR (internal standard: Nitromethane).

**Table 4:** Yield and conversion variation with time after stopping the electrolysis.

Time after Electrolysis/h	Yield of 3a ^a^ (%)	Conversion of 1a ^a^ (%)
0	13	47
1	18	53
2	21	58
3	24	61
5	24	61

^a^ Determined by RP-HPLC. Electricity for the electrolysis, 1 F·mol^−1^.

## Supplementary Materials

Supplementary materials are available online.
