# Supplementary material for: Cathodic Aromatic C,C Cross-Coupling Reaction via Single Electron Transfer Pathway
Source: Molecules. 2017 Mar 7;22(3):413. doi: 10.3390/molecules22030413 (PMC6155275; doi:10.3390/molecules22030413)
Supplement: Supplementary file 1 [file molecules-22-00413-s001.pdf]

Supplementary Materials: Cathodic Aromatic C,C Cross-Coupling Reaction via Single Electron Transfer Pathway

1. Linear Sweep Voltammograms for Reduction of Arylhalides and Arenes.

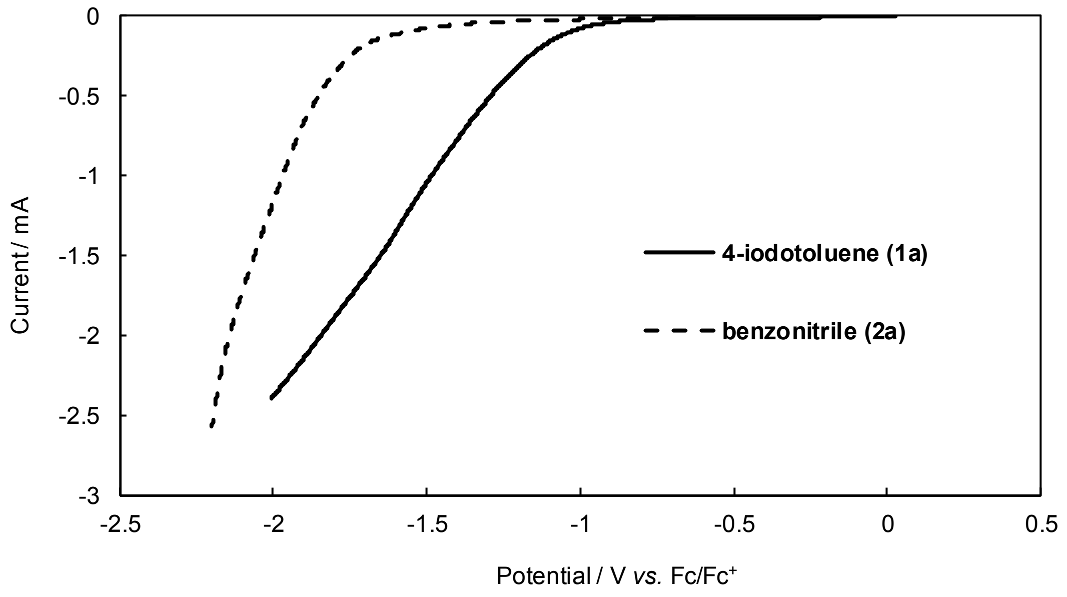

Figure S1. Linear sweep voltammograms of benzonitrile and 4-iodotoluene in DMF (10 mL) at 25°C at the scan rate 0.1 V s^{-1} on Pt electrode.

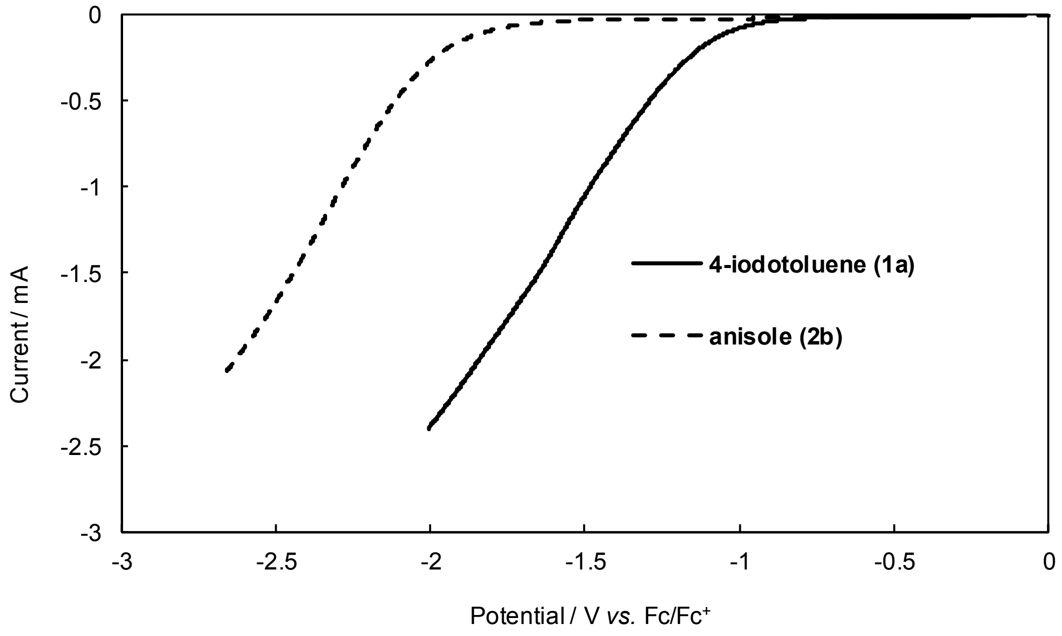

Figure S2. Linear sweep voltammograms of anisole and 4-iodotoluene in DMF (10 mL) at 25°C at the scan rate 0.1 V s^{-1} on Pt working electrode.

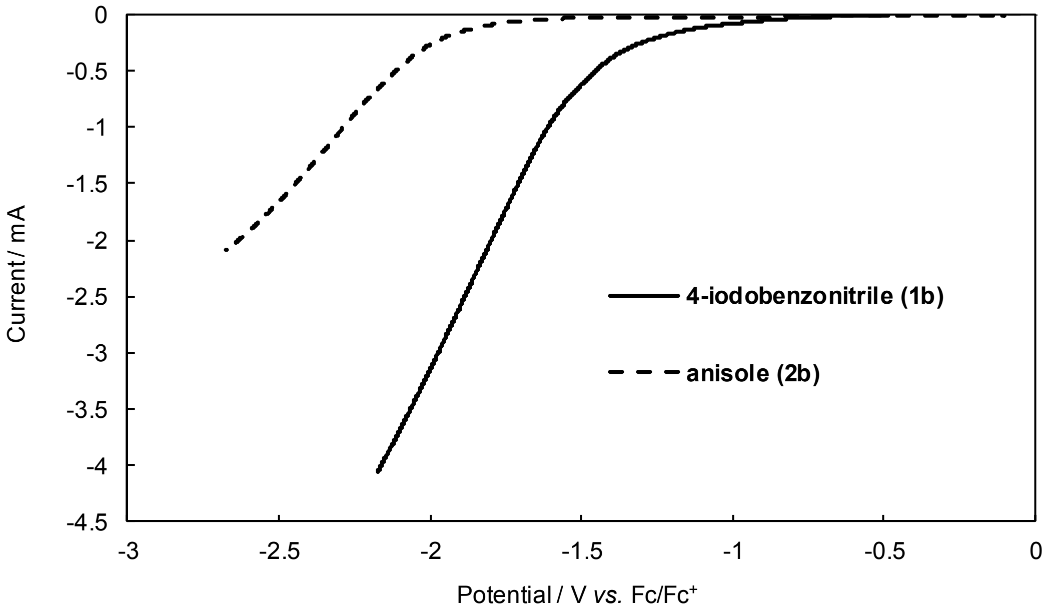

Figure S3. Linear sweep voltammograms of anisole and 4-iodobenzonitrile in DMF (10 mL) at 25°C at the scan rate 0.1 V s^{-1} on Pt working electrode.

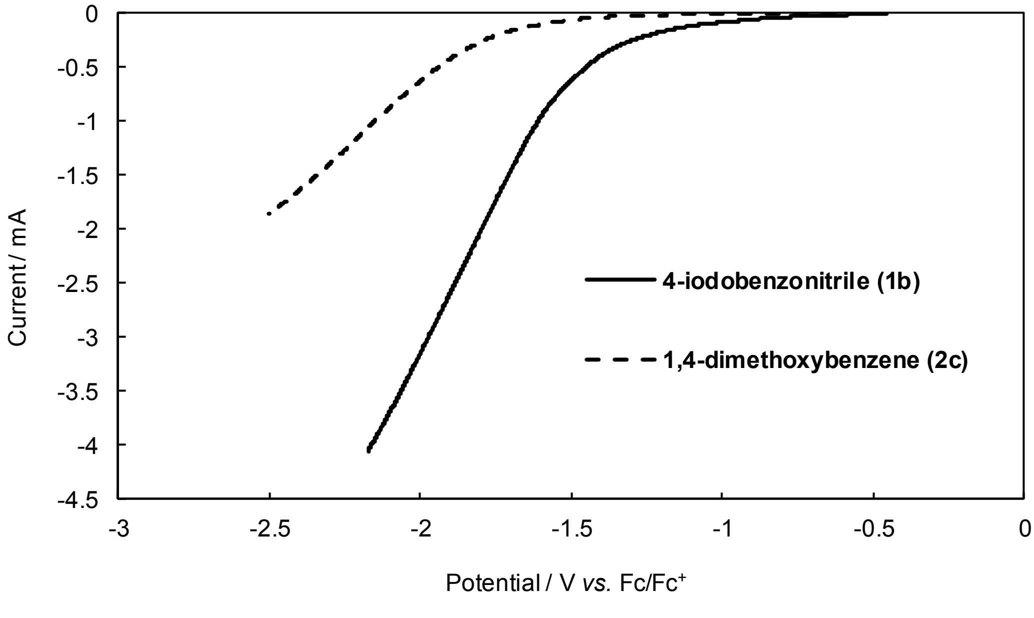

Figure S4. Linear sweep voltammograms of 1,4-dimethoxybenzene and 4-iodobenzonitrile in DMF (10 mL) at 25°C at the scan rate 0.1 V s^{-1} on Pt working electrode.

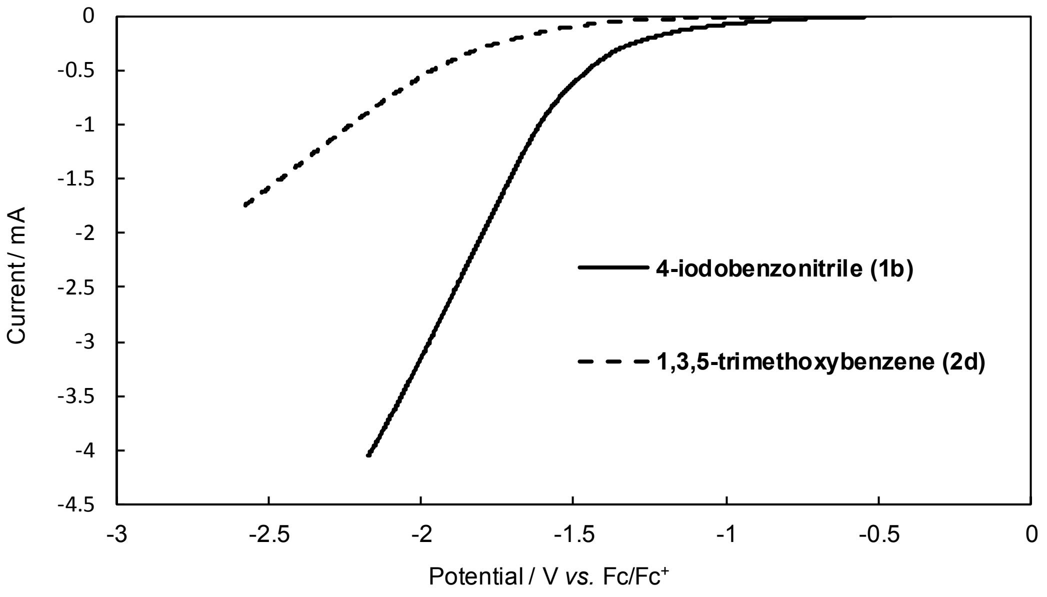

Figure S5. Linear sweep voltammograms of 1,3,5-trimethoxybenzene and 4-iodobenzonitrile in DMF (10 mL) at 25°C at the scan rate 0.1 V s^{-1} on Pt working electrode.

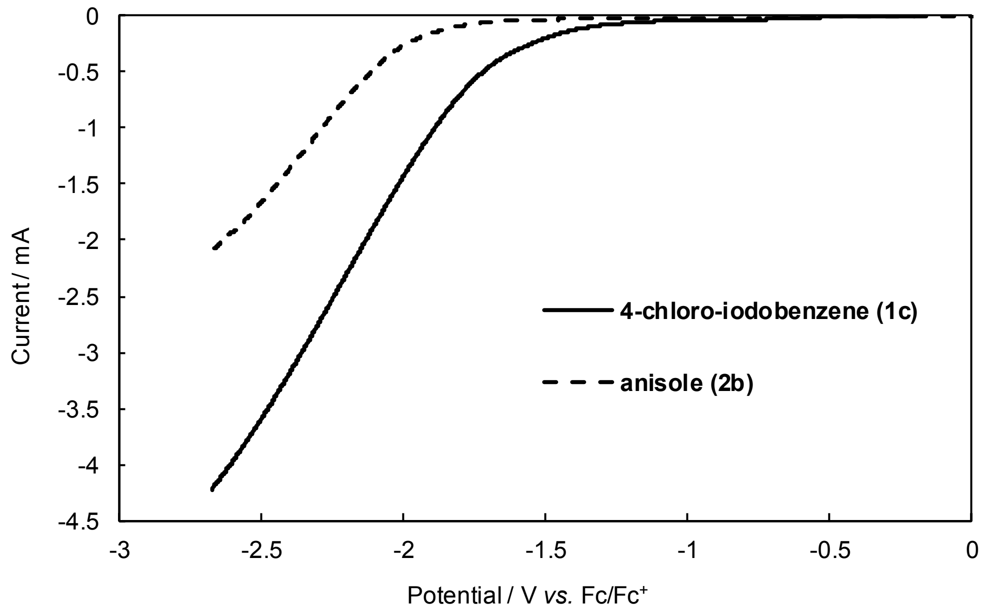

Figure S6. Linear sweep voltammograms of anisole and 4-chloro-iodobenzene in DMF (10 mL) at 25°C at the scan rate 0.1 V s^{-1} on Pt working electrode.

2. Determination of ^{1}H NMR Yields of 3b, 3c, 3d, and 3e

After the electrolysis, crude products were subjected to short silica gel column chromatography (solvent: chloroform) in order to remove supporting electrolytes. Then, the formation of cross coupling products were confirmed by capillary GC-MS analysis. The base peaks of **3b**, **3c**, **3d**, **3e**, and **3f** were observed at 198.05, 209.05, 239.05, 269.10, and 218.90, respectively. Nitromethane (0.75 μL, 0.014 mmol) as an internal standard was added to 0.2 mL of evaporated residues. The NMR yields of **3b**, **3c**, **3d**, and **3e** were determined by following equation (3).

$$I_1 / N_1 = I_2 / N_2 \quad —(1)$$
$$N_2 / 0.2 = N_3 / V —(2)$$
$$\text{Yield (\%)} = N_3 / N_{\text{arylhalide}} \cdot 100\% \quad —(3)$$

where I_1 and I_2 are NMR integral values of nitromethane and product peaks, respectively; N_1, N_2, and N_3 are mole quantities of nitromethane in NMR sample, product in 0.2 mL of evaporated residue, and product in total evaporated residue, respectively; V are volume of total evaporation residue. Details are shown in Table S1.

Table S1. Detail values for ^{1}HNMR yields determination

Cross-coupling product	I_1	I_2	V / mL	Yield (%)
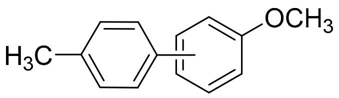 **3b**	10	o : m : p = 4,26 : 0 : 5.87	6.0	47 (o : m : p = 42 : 0 : 58)
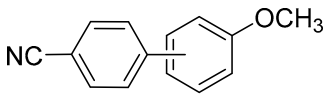 **3c**	10	o : m : p = 0 : 0 : 13.36	5.0	52 (o : m : p = 0 : 0 : 100)
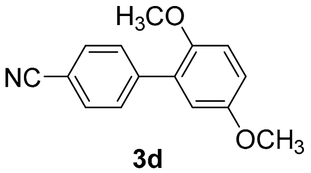 **3d**	10	8.94	6.0	42
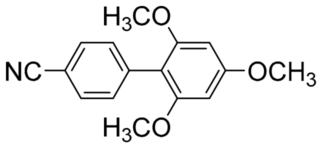 **3e**	10	6.82	4.0	11
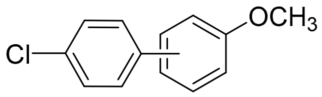 **3f**	10	o : m : p = 0 : 2.54 : 7.99	6.0	49 (o : m : p = 0 : 24 : 76)

3. Schematic illustration of H-type divided cell.

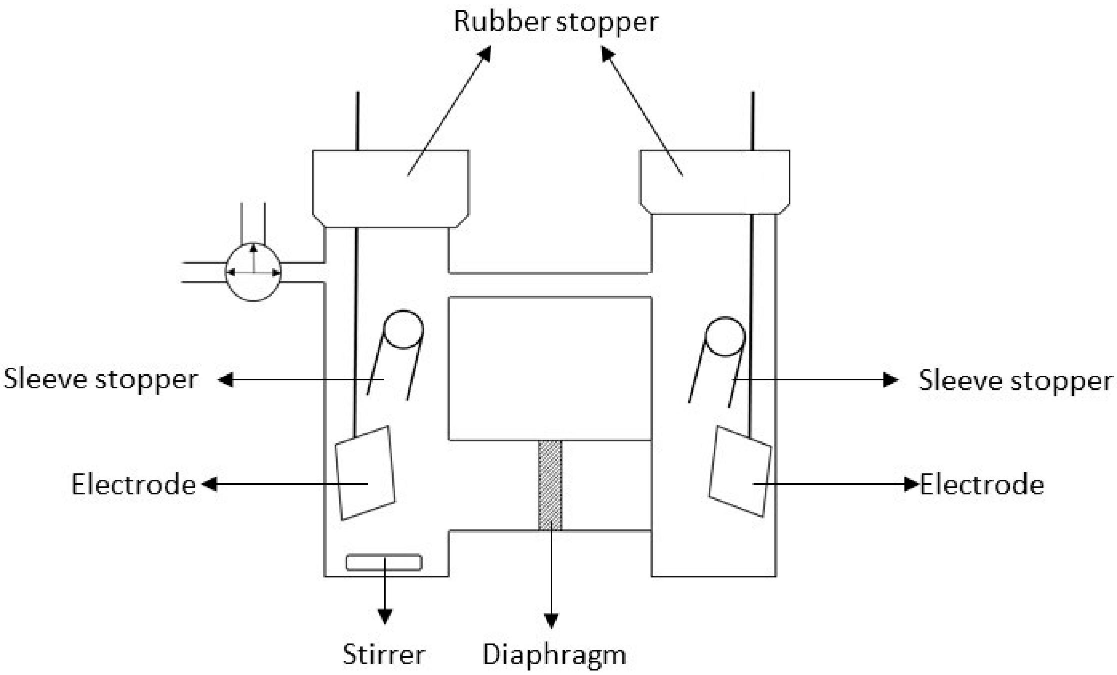

Figure S7. Schematic illustration of H-type divided cell.

4. ¹H NMR Spectra

The yields of **3b**, **3c**, **3d**, **3e**, and **3f** were calculated by ¹H NMR measurements of these reaction mixtures with nitromethane (at 4.30 ppm) as an internal standard. Characteristic peeks of purpose products were determined by referring to the previous synthetic reports [1-8].

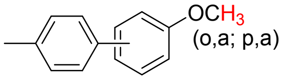

(**3b**)

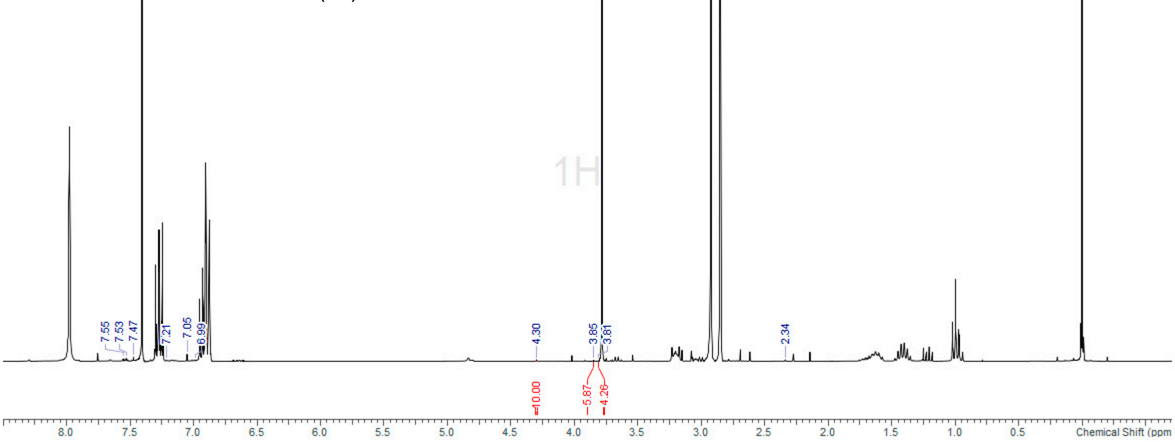

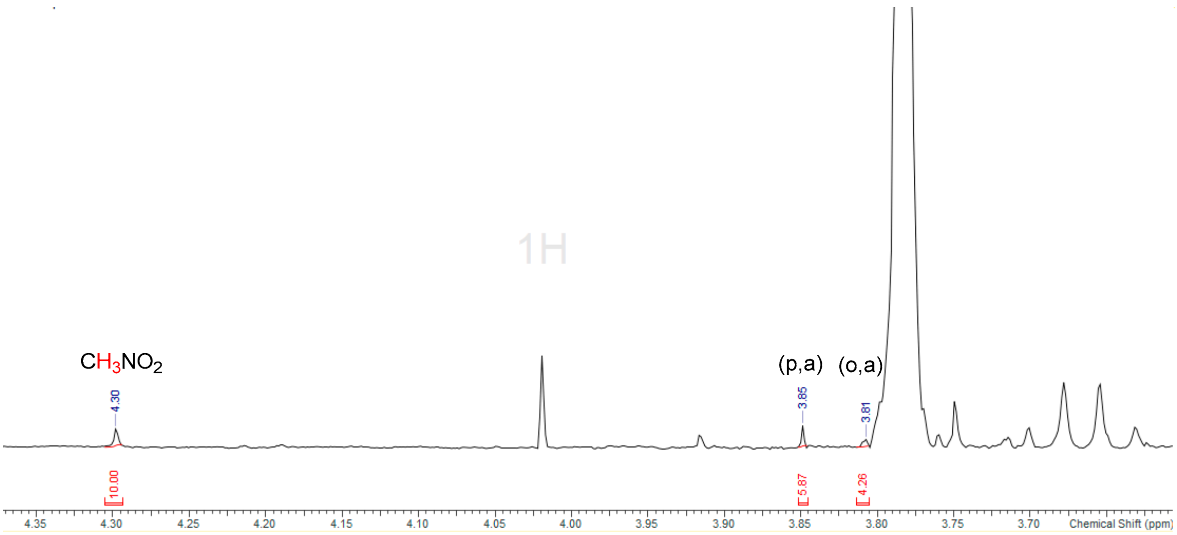

Figure S8. ^{1}H NMR spectrum of reaction mixture of Entry 2 in Table 3 (synthesis of **3b**).

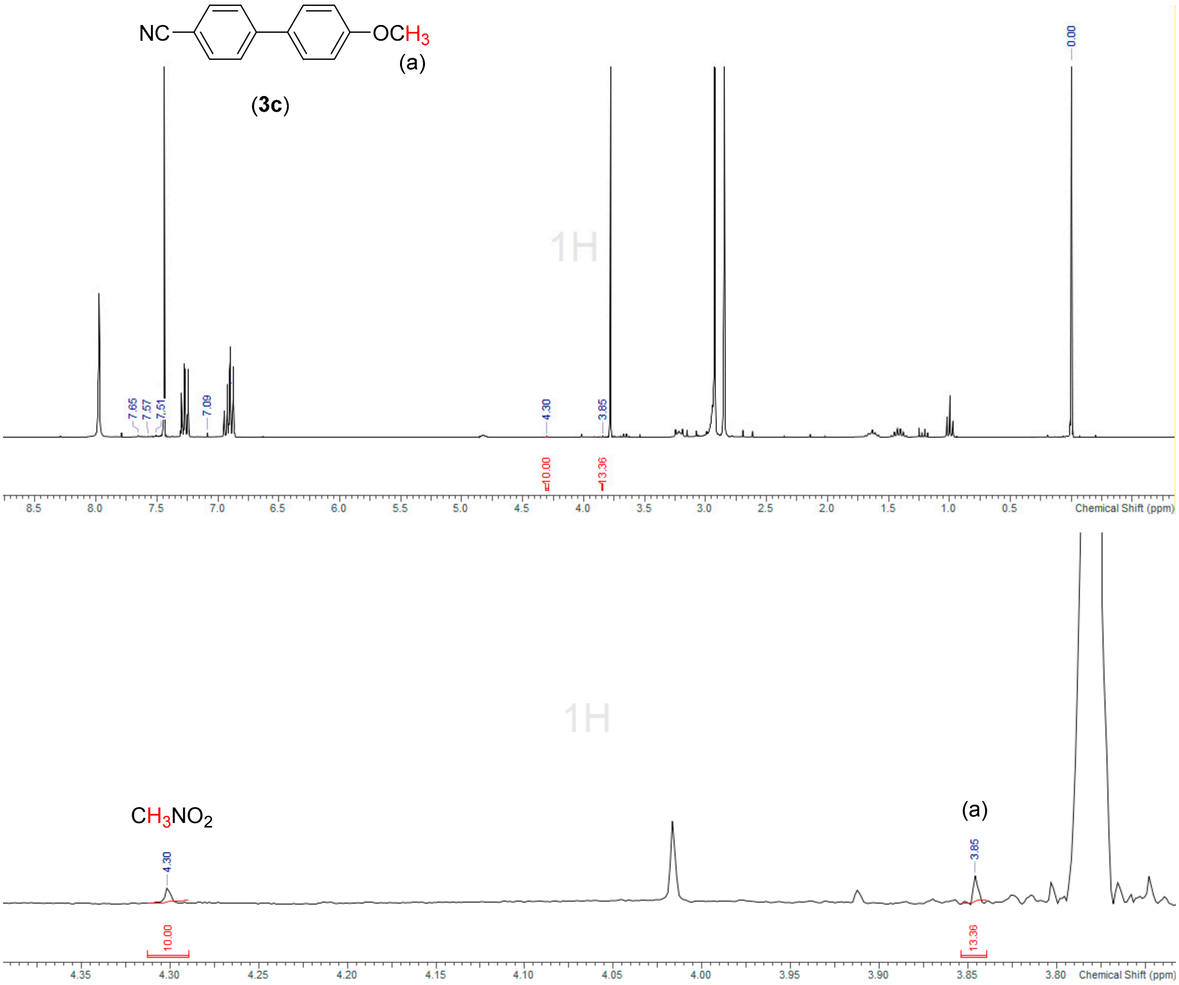

Figure S9. ^{1}H NMR spectrum of reaction mixture of Entry 3 in Table 3 (synthesis of **3c**).

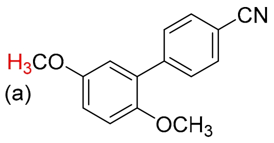

(**3d**)

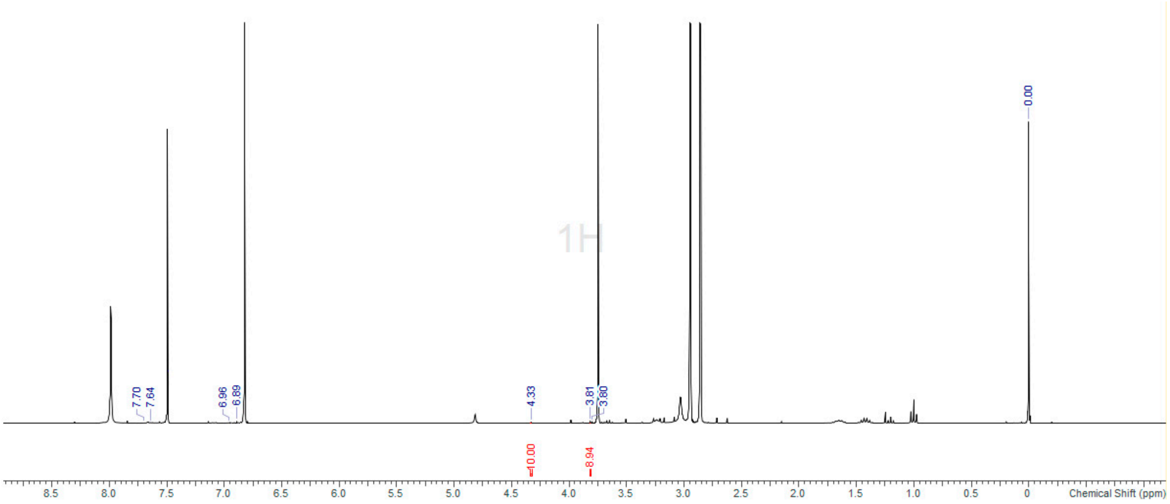

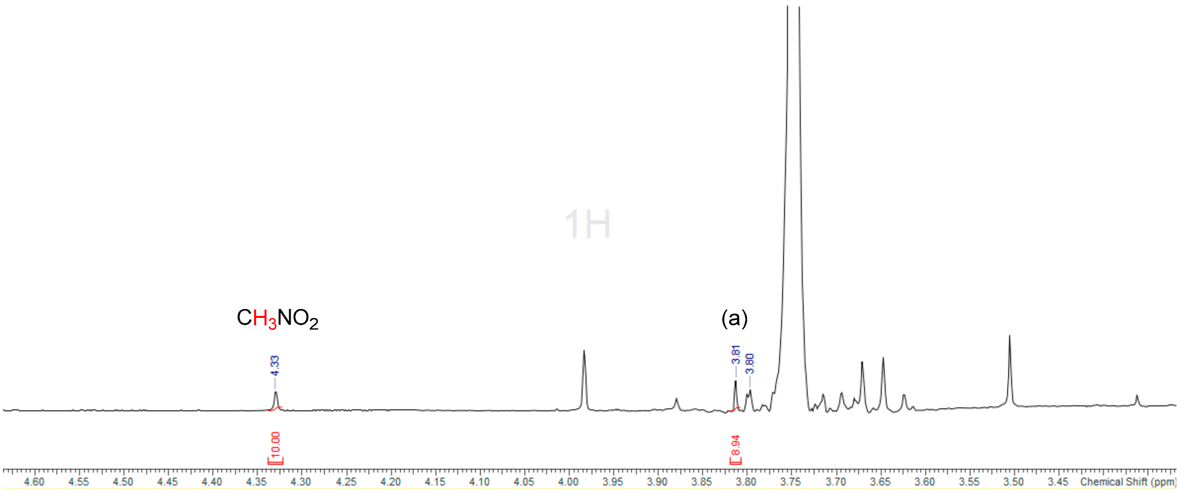

Figure S10. ^{1}H NMR spectrum of reaction mixture of Entry 4 in Table 3 (synthesis of **3d**).

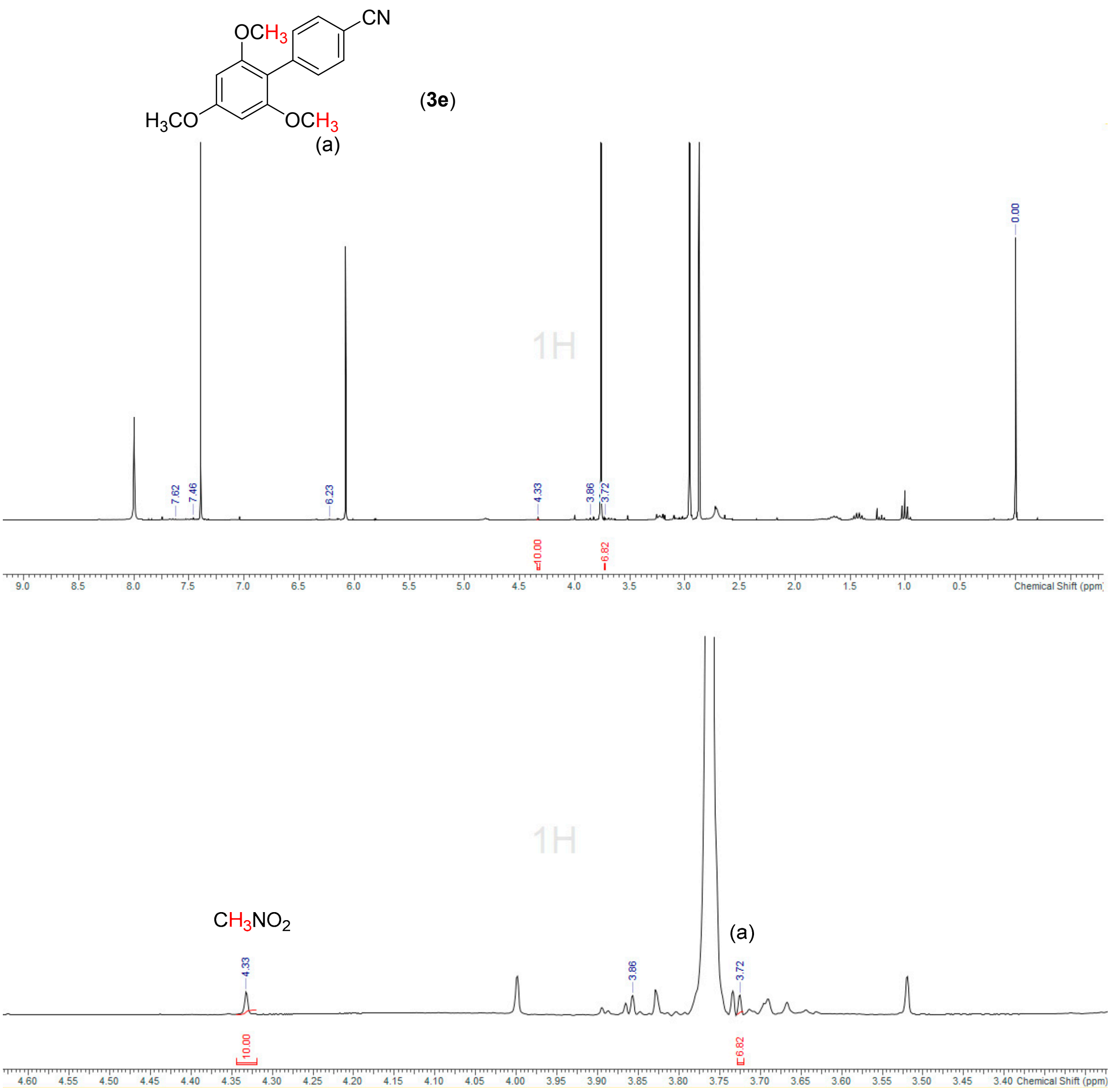

Figure S11. ^{1}H NMR spectrum of reaction mixture of Entry 5 in Table 3 (synthesis of **3e**).

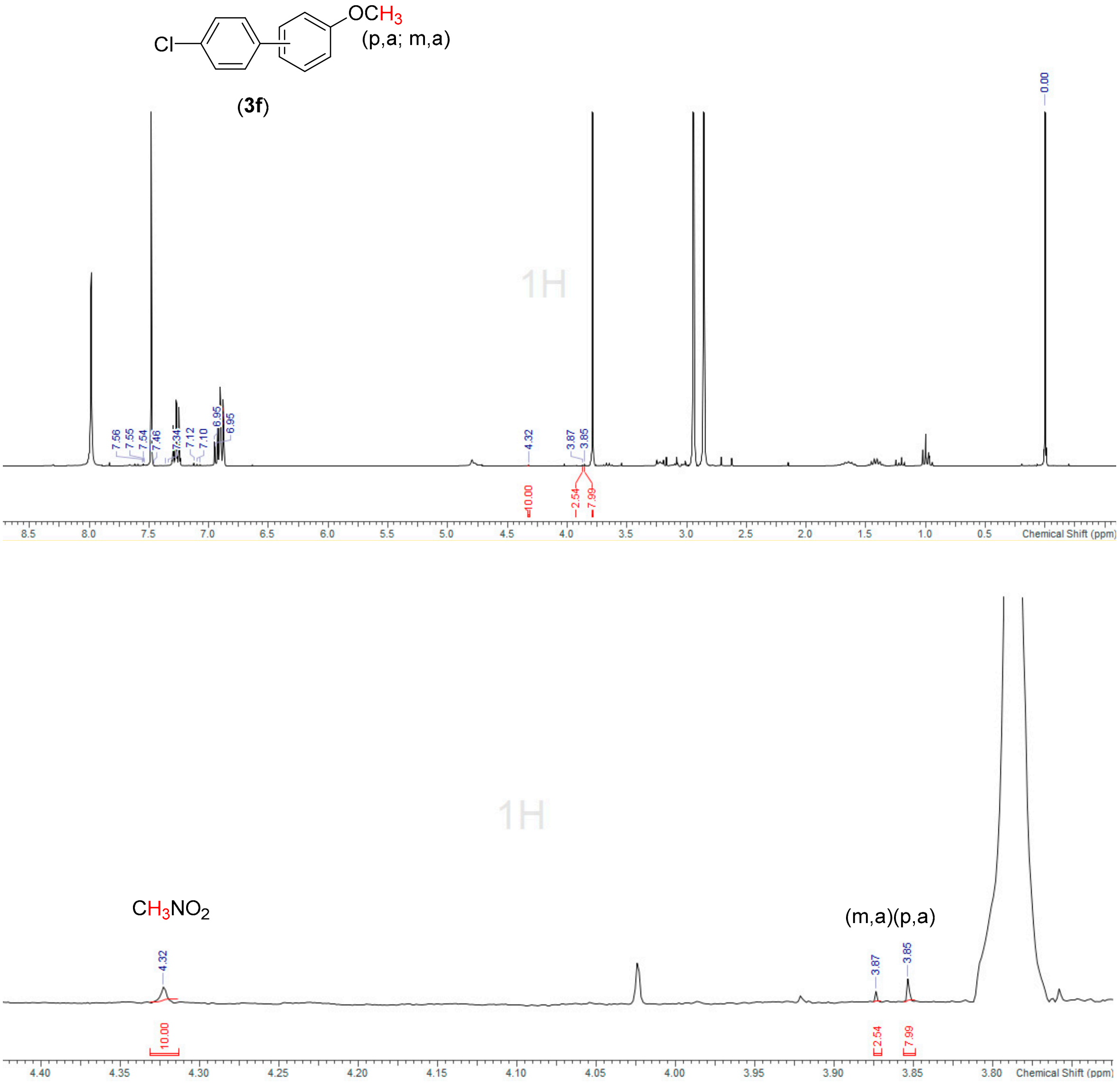

Figure S12. ^{1}H NMR spectrum of reaction mixture of Entry 6 in Table 3 (synthesis of **3f**).

References

1. Varnedoe, L.S.; Angel, B.D.; McClellan, J.L.; Hanna, J.M.Jr. Pd/C-catalyzed cross-coupling of arenediazonium salts with potassium aryltrifluoroborates. *Lett. Org. Chem.* **2010**, *7*, 1-6. [CrossRef][PubMed]
2. Yu, D.; Yu, M.; Guan, B.; Li, B.; Zheng, Y.; Wu, Z.; Shi, Z. Carbon-carbon formation via Ni-catalyzed Suzuki-Miyaura coupling through C-CN bond cleavage of aryl nitrile. *Org. Lett.* **2009**, *11*, 3374-3377. [CrossRef][PubMed]
3. Bernhardt, S.; Manolikakes, G.; Kunz, T.; Knochel, P. Preparation of solid salt-stabilized functionalized organozinc compounds and their application to cross-coupling and carbonyl addition reactions. *Angew, Chem. Int. Ed.* **2011**, *50*, 9205-9209. [CrossRef][PubMed]
4. Papoian, V.; Minehan, T. Palladium-catalyzed reactions of arylindium reagents prepared directly from aryl iodides and indium metal. *J. Org. Chem.* **2008**, *73*, 7376-7379. [CrossRef][PubMed]
5. Jasch, H.; Höfling, S.B.; Heinrich, M.R. Nucleophilic substitutions and radical reactions of phenylazocarboxylates. *J. Org. Chem.* **2012**, *77*, 1520-1532. [CrossRef][PubMed]

6. Dai, J.; Liu, J.; Luo, D.; Liu, L. Pd-catalysed decarboxylative Suzuki reactions and orthogonal Cu-based O-arylation of aromatic carboxylic acids. *Chem. Commun.* **2011**, *47*, 677-679. [CrossRef][PubMed]
7. Denmark, S.E.; Smith, R.C.; Chang, W.T.; Muhuhi, J.M. Cross-coupling reactions of aromatic and heteroaromatic silanolates with aromatic and heteroaromatic halides. *J. Am. Chem. Soc.* **2009**, *131*, 3104-3118. [CrossRef][PubMed]
8. Ackermann, L.; Althammer, A. Air-stable PinP(O)H as preligand for palladium-catalyzed Kumada coupling of unactivated tosylates. *Org. Lett.* **2006**, *8*, 3457-3460. [CrossRef][PubMed]
